# Retraction Note: Noise tailoring for quantum circuits via unitary 2*t*-design

**DOI:** 10.1038/s41598-023-40529-3

**Published:** 2023-08-15

**Authors:** Linxi Zhang, Yan Yu, Changhua Zhu, Changxing Pei

**Affiliations:** 1 State Key Laboratory of Integrated Services Networks, Xidian University, Xi’an, 710071 China; 2 Science and Technology on Communication Networks Laboratory, Shijiazhuang, 050081 China

Retraction of: *Scientific Reports* 10.1038/s41598-018-38158-2, published online 11 February 2019

The Editors have retracted this Article.

Technical issues have been identified in the proofs of Theorems 1 and 2. Equation (17) is incorrect, since it should be the average over all pure input states and not only over tensor product states. This issue invalidates the proof of Theorem 1, because the quantity being evaluated cannot be reduced to a twirled channel acting on a single input state as claimed. In Theorem 2, local random circuits are used to construct a unitary 2t-design. However, since only separable noise channels are considered, this result is just a special case of Ref. #12. As Theorems 1 and 2 are the main results of the study, the Editors no longer have confidence in the results and conclusions presented.

Linxi Zhang and Yan Yu have not responded to correspondence from the Editors about this retraction. The Editors were not able to confirm the current contact details for Changhua Zhu and Changxing Pei.

